# Mental Status as a Common Factor for Masticatory Muscle Pain: A Systematic Review

**DOI:** 10.3389/fpsyg.2017.00646

**Published:** 2017-05-09

**Authors:** Mieszko Wieckiewicz, Marek Zietek, Joanna Smardz, Dobrochna Zenczak-Wieckiewicz, Natalia Grychowska

**Affiliations:** ^1^Department of Prosthetic Dentistry, Wroclaw Medical UniversityWroclaw, Poland; ^2^Department of Periodontology, Wroclaw Medical UniversityWroclaw, Poland; ^3^Department of Oral Surgery, Wroclaw Medical UniversityWroclaw, Poland

**Keywords:** myofascial pain syndrome, masticatory muscle pain, masseter muscle pain, mental disorders, anxiety, depression, mood disorders, stress-related disorders

## Abstract

Masticatory muscle pain (MMP) is the primary reason for chronic non-odontogenic orofacial pain in the human population. MMP has become a considerable social problem, which affects about 12–14% of the adult population and is 1.5–2 times more frequent in women than in men. This term defines a pain which has its origins in the masticatory muscles. Although MMP is typically felt in the face, jaws, and preauricular area, MMP can radiate to the ear, teeth, head, and neck. This systematic review explains the relationship between MMP and common mental states, such as anxiety, depression, mood and stress-related disorders, and is reported in accordance with PRISMA guidelines. We performed a search in the PubMed database for peer-reviewed articles published after November 1st 2006 in the context of MMP and mental states. According to the defined criteria, 38 studies were finally included into the systematic review, of which prospective cohort studies were found to be the most common. We investigated four primary outcomes (anxiety, depression, mood disorders, and stress-related disorders) and several secondary outcomes of search. Seventy-nine percent of studies concerned depression, 42% anxiety, 29% mood disorders, and 21% stress-related disorders. Most of the studies showed a relationship between MMP and alterations in mental status. Nonetheless, the researchers usually evidenced only the co-occurrence of psychiatric disorders and dysfunctions of the masticatory muscles among the group of patients, in large part in women. Moreover, some studies were marked with limited generalizability of the reported results, quality flaws and heterogeneity. In the light of the analyzed literature, the causal relationship between mental states and MMP is still not clearly established.

## Introduction

A number of papers confirm that there is a correlation between pain sensitivity and mental states (Gatchel, 2004; Means-Christensen et al., 2008; Vaccarino et al., 2009; Haviland et al., 2011). Moreover, muscle pain also seems to be closely involved in this pathomechanism (Rollman and Gillespie, 2000; Haviland et al., 2011; Rees et al., 2011; Hung et al., 2016). Based on the previously cited papers (Rollman and Gillespie, 2000; Gatchel, 2004; Means-Christensen et al., 2008; Vaccarino et al., 2009; Haviland et al., 2011; Rees et al., 2011; Hung et al., 2016), this pathomechanism usually involves back, neck and orofacial muscles including masticatory muscles. Defining the onset of orofacial muscle pain related to psychoemotional status is controversial. Therefore researchers are still looking for a clear explanation of this important clinical issue (Durham et al., 2015; Glaros et al., 2016).

According to the literature, the term masticatory muscle pain (MMP) describes “the pain with origin in the masticatory muscles, including tendons and fasciae” and “is diagnosed by the presence of tenderness to palpation, e.g., of tender but not trigger points” (Gatchel, 2004). Alternatively, in the commonly applied research diagnostic criteria for temporomandibular disorders (RDC/ TMD) to define the same muscle condition, the term myofascial pain (MFP) is used. Interestingly, MFP is used to define the pain caused by trigger points (Gatchel, 2004). In our study, we consider these both symptoms of muscle pain dysfunction, i.e., related to trigger points and tenderness. MMP diagnosed based on muscle tenderness with palpation occurs in 12–14% of the examined population—there is a 1.5–2 times higher chance that women will suffer from this medical condition than men (Gatchel, 2004). Surprisingly, the prevalence of MMP in the age range 7–17 years is not higher in girls than boys (Gatchel, 2004).

The etiology and likely mechanism of muscle pain has aroused many controversies over the years. It can be associated with a peripheral mechanism of muscle pain excitation, outlasting sensitization of peripheral nociceptors, which are involved in the excitation of central neurons, and/or functional disorders (Haviland et al., 2011). However, numerous sources indicate that, regardless of the original muscle pain pathology, excessive muscle tension simultaneously appears as defensive muscle reaction, and results in increasing intensification of symptoms (Means-Christensen et al., 2008; Haviland et al., 2011). Increased muscle tension can be a local result of trauma, physiological function or dysfunction, and a defensive response to psychological burden (Means-Christensen et al., 2008; Haviland et al., 2011). The central mechanism of muscle pain development due to long-term overactivity mostly relates to those muscles which tense during psychological discomfort, anxiety, anger, and bad mood or under stress (Vaccarino et al., 2009; Haviland et al., 2011). The masseter, temporal muscle, sternocleidomastoid muscle and trapezius muscle, especially its upper part, are very good examples of mentioned central mechanism. These muscles tense in response to alterations in mental status, which may induce muscle pain related to mental disorders or chronic stress (Hung et al., 2016).

According to Okeson, the MMP can be divided into several types: protective co-contraction, local muscle soreness, MFP, myospasm (tonic contraction myalgia) and centrally mediated myalgia (Okeson, 2013). In many cases, the risk of the development of these MMP types is centrally modulated and is dependent on the mental state of the patient. Moreover, there are great similarities, and possible overlaps, between patients suffering from MMP and tension-type headache (TTH) and/or fibromyalgia (FM) (Rollman and Gillespie, 2000). A muscular pain was a cause of a headache in about 38% of adults (Rees et al., 2011).

We try to explain the relationship between MMP and psychological alterations and/or disorders such as anxiety, depression, mood and stress-related disorders, as well as systematically reviewing the current literature related to this issue and investigating it objectively.

## Materials and methods

We followed the Preferred Reporting Items for Systematic Review and Meta-Analysis (PRISMA) guidelines for our systematic review and to collect and report data (Moher et al., 2009; Shamseer et al., 2015).

### Eligibility criteria for initial study selection

#### Studies

We established the following inclusion criterion: clinical trials or experimental studies, which concern relationships between MMP and common mental states, such as anxiety, depression, mood disorders and stress-related disorders. Peer-reviewed, English-language and full text articles published after November 1st 2006 were included into our study. Articles which discussed the research topic only in the introduction or discussion were excluded.

#### Participants

Participants were males and females of any age with clinical diagnosis of MMP and/or TTH accompanied by psychological variables and/or mental disorders.

#### Outcomes

A study was included into this systematic review if it investigated at least one of the primary outcomes of interest: anxiety, depression, mood and stress-related disorders. Moreover, secondary outcomes such as pain, patient's quality of life, sleep disturbances, and somatization were also considered.

### Data sources and searches

We searched the PubMed database to identify relevant publications. To make the process of searching more efficient, we added PubMed filters to find clinical trials, meta-analyzes, randomized controlled trials, and systematic reviews. Additional filters included articles published after November 1st 2006, available in English. Medical subject headings (MeSH) were used to develop a literature search strategy (Moher et al., 2009; Shamseer et al., 2015) as follows: each of three synonymous phrases, i.e., (1) *masticatory muscle pain*, (2) *masticatory myofascial pain*, and (3) *myofascial pain syndrome*, were combined with each mental status: (a) *anxiety*, (b) *depression*, (c) *mood disorders*, and (d) *stress*-*related disorders*, e.g., “masticatory muscle pain anxiety,” viz. (1)+(a); “masticatory muscle pain depression,” viz. (1)+(b); “masticatory myofascial pain mood disorders,” (2)+(c), etc. In this way, we obtained 12 queries. We also screened the reference list of included studies to trace potentially relevant papers.

### Trial selection

Three authors were involved in the literature screening procedure (MW, JS, and NG). Firstly, the titles, abstracts and full texts were screened independently by two of these authors (JS and NG). Then, the full texts of potentially suitable articles were screened for key words, such as “*masticatory*,” “*myofascial*,” “*orofacial*,” “*facial*,” “*pain*,” “*temporomandibular*,” “*masseter*,” “*temporalis*” “*myogenous*,” “*muscle*,” “*anxiety*,” “*depression*,” “*stress*,” and “*mood*,” and the articles were then evaluated for their relevance in the context of the research question. The next step of paper selection involved a final evaluation of relevance and practical validity regarding the relationship between mental status and MMP. Eventually, the three authors jointly decided whether the identified articles met the inclusion criteria. Neither of the review authors was blind to the journal title or to the study authors or institutions (Tricco et al., 2012; Shamseer et al., 2015).

### Data extraction

After the final agreed decision, two reviewers conducted data extraction independently (JS, NG). Then, the third author (MW) checked the validity of all data extracted. The extraction process included information regarding study design, sample characteristics and size, diagnostics criteria, comparison groups, outcomes and tools used to measure those outcomes.

### Data synthesis and analysis

The studies included in our systematic review are very heterogeneous. As a consequence, performance of a meta-analysis was impossible. We conducted a “narrative, qualitative summary” as recommended (Mao et al., 2015). The quality evidence for results was assessed employing the Grading of Recommendations Assessment, Development and Evaluation working group approach (Young et al., 2012; Shamseer et al., 2015)^1^. The quality evidence for each outcome was assessed as one of the following categories: very low, low, moderate or high.

## Results

### Description of studies

**Figure 1:**
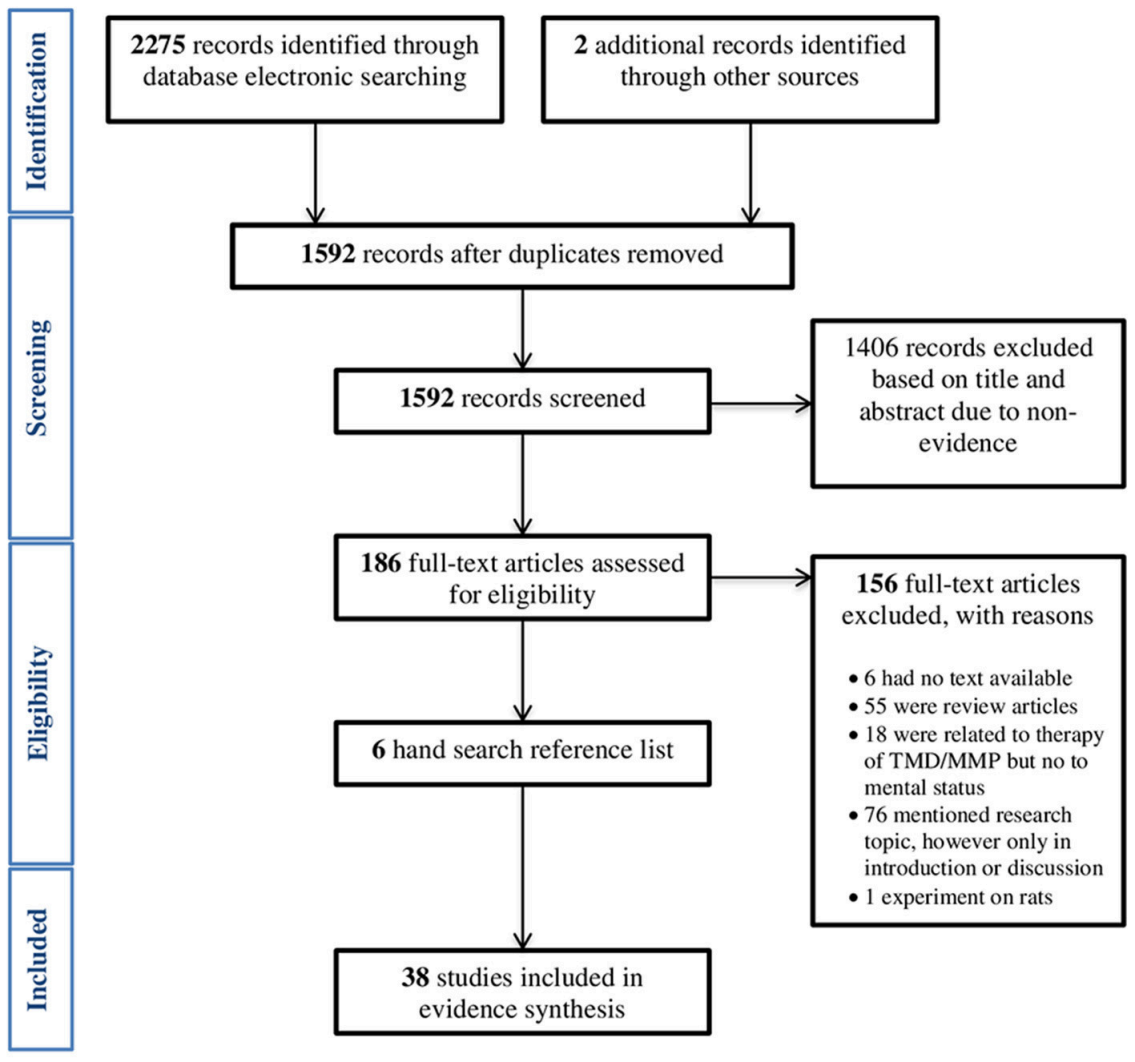
**Flow diagram of the systematic review protocol**.

**Figure 2:**
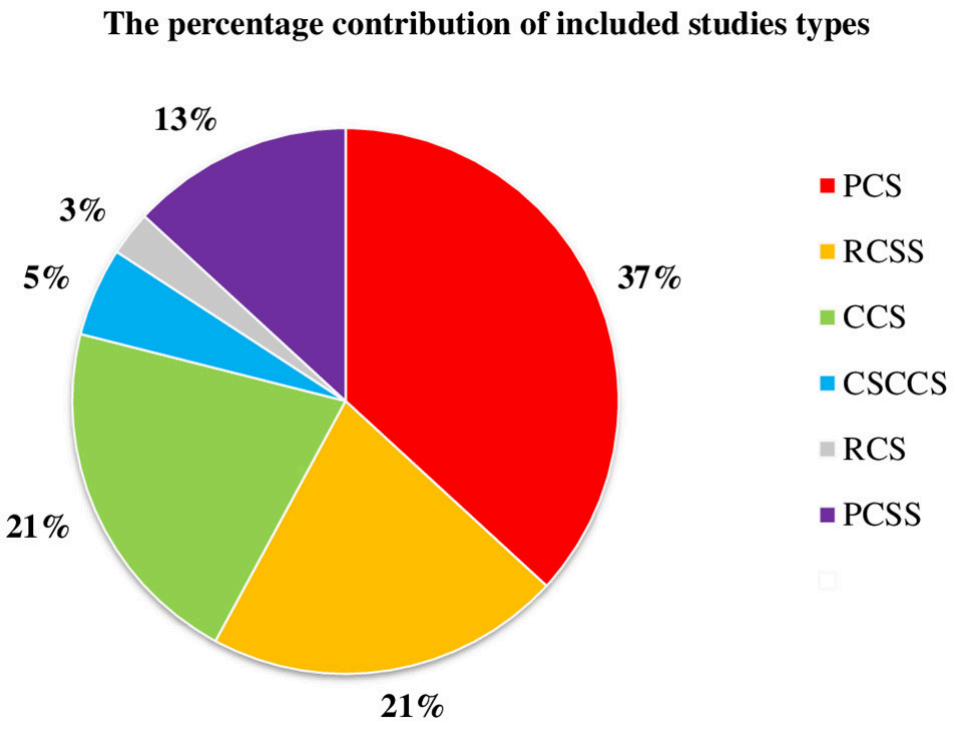
**The percentage contribution of included studies types**. PCS, prospective cohort study; RCSS, retrospective cross-sectional study; CCS, case-control study; CSCCS, cross-sectional case-control study; RCS, retrospective cohort study; PCSS, prospective cross-sectional study.

According to the above search protocol, we obtained 2,275 results from searches conducted up to and including October 2016. Thirty-nine articles were included. Although, we searched for clinical trials, meta-analyzes, randomized controlled trials, and systematic reviews, we did not find such types of studies. The main reasons for exclusion were study design (review articles and not systematic reviews), studies' relationship to the therapy of TMD (not accompanied by mental status reports), and mention of the research topic, however only in the introduction or discussion. The systematic review protocol is presented as a flow-chart in Figure 1. Most studies confirmed the relationship between MMP and at least one mental state, but there were also two studies that definitely did not support any of mentioned assertions (Nilsen et al., 2008; Calixtre et al., 2014). Qualified research in the majority of cases described the co-existence of MMP and mental disorders, but did not indicate their cause and effect relationship. The percentage contribution of the particular research designs is displayed in chart form (Figure 2). Another interesting issue to mention is that the problem was associated mainly with female gender. An overview of the content of qualifying studies is presented in Table 1.

**Table 1:** **An overview of the content of qualified studies**.

**Authors (year)**	**Studied mental states**	**Study designs**	**Sample size**	**Diagnose criteria**	**Outcomes**	**Tools used to measure outcomes**
Calixtre et al. (2014)	Anxiety, depression	PCS	*N* = 19 (95% female)	RDC/TMD	Anxiety and depression scores did not change clinical symptoms or jaw functionality in college students suffering from TMD	Clinical evaluation according to RDC/TMD; MFIQ; HADS; EMG
Nilsen et al. (2008)	Stress-related disorders	PCS	*N* = 18 (100% females)	The ACR criteria for FM; Diagnose of chronic shoulder/neck pain	The reduction of sympathoneural input to the shoulder/neck region did not impact the pain, neither muscular response to low-grade experimentally induced mental stress among FM patients	VAS to report pain, perceived tension, and fatigue; EMG
Slade et al. (2007)	Depression, Mood disorders, Stress-related disorders,	PCS	*N* = 171 (100% females)	RDC/TMD (excluding criterion)	“Depression, perceived stress, and mood were associated with pain sensitivity and were predictive of 2- to 3-fold increases in risk of TMD”	Clinical evaluation according to RDC/TMD; COMT genotyping, psychological questionnaires; psychophysical pain assessments
Vedolin et al. (2009)	Anxiety, Stress-related disorders	CCS	*N* = 48 (100% females)	RDC/TMD	During stressful periods the pressure pain thresholds among MFP subjects were significantly lower than in controls. No difference between groups in anxiety and stress at any time	Clinical evaluation according to RDC/TMD; Beck Anxiety Inventory, Lipp Stress Symptoms Inventory; VAS to report pain intensity; PPT determining with electronic algometer
García-Moya et al. (2015)	Anxiety	CCS	*N* = 38 (100% females)	ACR diagnostic criteria for FM	Higher prevalence of masseter pain under palpation, also headache/neck pain and stiff/tried jaw among FM patients. Comparison between FM cases and healthy controls in anxiety was no significant. Intensity of pain and state anxiety index was correlated	A self-administered TMD screening questionnaire recommended by the AAOP; physical examination, VAS to report intensity of pain, STAI
Nilsson and Dahlström (2010)	Mood disorders, Stress-related disorders	CCS	*N* = 60 (100% females)	RDC/TMD	Higher emotional distress, fatigue, cognitive difficulties were among patients with TMDs, than among healthy controls. No differences in salivary cortisol measurements between groups	Clinical examination in accordance with RDC/TMD; VAS to report pain; Swedish version of the Profile of Fatigue Related Symptoms; salivary cortisol level assays
Park et al. (2010)	Depression, somatization	CCS	*N* = 75 (81% females)	RDC/TMD GCPS	Significantly higher depression scores in the myogenous pain subgroup of TMD patients. Pain intensity correlated with somatization and depression	Clinical evaluation according to RDC/TMD; GCPS; Thermosensory Analyzer II; SCL-90-R
Velly et al. (2011)	Depression, mood disorders	PCS	*N* = 480	CMI/RDC GCPS	Positive correlation between the intensification of pain and catastrophizing or depression	BDI; GCPS; CSQ; CMI
Kindler et al. (2012)	Anxiety, depression	PCS	*N* = 4308	Guidelines for diagnosis of TMD by the AAOP	Depression caused an increased risk of TMD joint pain upon palpation. Anxiety symptoms were correlated with joint and muscle pain	Clinical evaluation according to guidelines for diagnosis of TMD by the AAOP; self-administered health- and risk-factor-related questionnaire; CID-S
Boggero et al. (2016)	Depression	RCSS	*N* = 343 (82% females)	AAOC	Satisfaction with life in masticatory MFP patients was correlated with affective distress.	The Satisfaction With Life Scale; pain duration; VAS to record pain intensity; The West Haven- Yale Multidimensional Pain Inventory; The Multidimensional Fatigue Inventory–Short Form
Pfau et al. (2009)	Anxiety, depression, mood disorders	CCS	*N* = 55 (87% females)	RDC/TMD category I; ACR diagnostic criteria for FM	Higher levels of pain, catastrophizing, pain disability (but no anxiety and depression) were found in TMD patients compared to healthy individuals	Clinical evaluation according to RDC/TMD and ACR diagnostic criteria; Tender point score; PTT sum score; Quantitative sensory testing; German version of HADS, Pain Disability Index, CSQ
Velly et al. (2010)	Depression	PCS	*N* = 572 (88% females)	ACR diagnostic criteria for FM	The amplification of pain symptoms from mild to moderate or severe TMJD pain and disability was associated with FM, widespread pain, and depression	Clinical evaluation according to CMI/RDC; GCPS, Rheumatic Problems Questionnaire; tender-point examination for FM diagnose according to ACR; BDI
Alfvén (2012)	Stress-related disorders	PCS	*N* = 47 children	Diagnosis of FM	The specific pattern of stress tender points was different from that found in FM. Stress tender point localized near the muscle-tendon junction in the temporal region was found only in children suffering from prolonged stress	Questions about intensity, duration and frequency of pain; clinical examination of tender points
de Tommaso et al. (2009)	Anxiety, depression	PCSS	*N* = 217 (79% females)	ICHD-II	Higher anxiety and depression levels in patients with FM comorbidity, especially in migraine and tension-type headache sufferers	Total Tenderness Score, Zung Self-Rating Depression and Anxiety Scales, Migraine Disability Assessment Scale, Short Form 36 Health Survey and Medical Outcomes Study-Sleep Scale, Multidimensional Assessment of Fatigue, the Pain VAS, Manual Tender Point Survey and Fibromyalgia Impact Questionnaire
Mongini et al. (2007)	Anxiety, depression	RCSS	*N* = 649 (82% females)	Guidelines of the ICHD-II; DSM-IV	Higher prevalence of anxiety in myogenous pain patients. Regardless of the diagnostic group, anxiety and depression increased muscle tenderness score	Clinical examination according to ICHD-II and AAOP; pericranial, cervical, and cumulative tenderness scores
Glaros et al. (2007)	Stress-related disorders	CCS	*N* = 40 (88% females)	RDC/TMD; Diagnosis of chronic headache according to the IHS	Headache patients had more frequent diagnosis of MFP, masticatory muscle tension, more stress and more pain in the face/head than non-headache controls	Structured interview for the headache subjects; clinical examination in accordance with RDC/TMD; numerical rating scales to report pain, tension in the jaw, face or head, mood, and stress
Vidaković et al. (2016)	Depression, stress-related disorders	CCS	*N* = 101 (0% females)	RDC/TMD DSM-IV	“Higher severity of depression was accompanied by a higher percentage of subjects with MFP”	Clinical evaluation according to RDC/TMD; anamnestic history
Penna et al. (2009)	Depression, mood disorders	PCS	*N* = 60 (68% females)	CMI	Psychopathological aspects such as depression increased muscle tenderness and pain	CMI; SCID
Manfredini et al. (2011)	Anxiety, depression, mood disorders	PCS	*N* = 15 (47% females)	RDC/TMD (excluding criterion)	Masticatory muscle activity was related to trait anxiety scores, but not to anxiety state, depression or anger	Clinical evaluation according to RDC/TMD; Italian version of General Health Questionnaire; n EMG; STAI X-form; State-Trait Anger eXpression Inventory; BDI
Komiyama et al. (2008)	Anxiety	PCS	*N* = 24 (50% females)	RDC/TMD (excluding criterion)	State anxiety influenced electrical reflex threshold and electrical pain threshold of the masticatory muscles	Clinical evaluation according to RDC/TMD; EMG; STAI; pressure algometer
Klasser et al. (2014)	Anxiety depression, phobia	RCSS	*N* = 274 (85% females)	RDC/TMD	Increased severity of pain, anxiety, depression, psychiatric treatment, phobias, and severe headaches among patients with myogenous TMD	Clinical evaluation according to RDC/TMD; general medical health questionnaire; numerical rating pain scale
Pizolato et al. (2013)	Anxiety, depression	PCS	*N* = 82 (51% females)	RDC/TMD	TMD was significantly correlated with anxiety in children	Clinical evaluation according to RDC/TMD; HADS
Nifosì et al. (2007)	Anxiety, depression, mood disorders	PCSS	*N* = 63 (75% females)	RDC/TMD	MFP was correlated with anxiety, paranoia, psychoticism, hostility subscales, and psychological distress	Clinical evaluation according to RDC/TMD; SCL-90R; HDRS; HARS; Global Severity Index; anamnestic psychiatric information
Galli et al. (2009)	Anxiety, depression	CSCCS	*N* = 40 (85% females)	RDC/TMD category I	After administration of dexamethasone, the decrease in the cortisol levels in the myogenous pain patients was significantly larger than in the control group. The myogenous pain patients exhibited significantly higher scores on depression, anxiety, physical fatigue and mental fatigue	Clinical evaluation according to RDC/TMD; German version of the HADS Scale; the Fatigue Scale; VAS to assess pain, sleep duration and quality; dexamethasone suppression test; salivary cortisol assays
Nadendla et al. (2014)	Anxiety	PCSS	*N* = 20 (55% females)	RDC/TMD	A positive correlation between anxiety and the salivary cortisol levels in MFP patients	Clinical evaluation according to RDC/TMD; HDRS, salivary cortisol level assays
Giannakopoulos et al. (2010)	Anxiety, Depression	CCS	*N* = 222 (73% females)	RDC/TMD; diagnosis of chronic facial pain other than TMD	Higher depression levels among MFP patients than in the general population. No significant effect for sex or TMD subgroup for anxiety	Clinical examination in accordance with RDC/TMD; German version of the HADS
Guarda-Nardini et al. (2012)	Anxiety, depression	RCSS	*N* = 110 (81% females)	RDC/TMD	Intensity of pain associated with depression and anxiety. No differences between patients with pain in jaw muscles and/or joint	Clinical evaluation according to RDC/TMD; pain diffusion, location, duration questionnaires; VAS to report pain intensity; HDRS; HARS; SCL-90R
Cioffi et al. (2014)	Depression, somatization	PCS	*N* = 781 (78% females)	RDC/TMD	No differences between groups of patients (myofacial pain, migraine, both myofascial pain and migraine) in depression scores. Muscular pain and migraine influenced the psychological status and determined higher somatization scores	Clinical evaluation according to RDC/TMD; clinical examination; IHS criteria; SCL-90; GCPS
Ivkovic et al. (2008)	Depression	RCSS	*N* = 68 (56% females)	RDC/TMD	Long-term, combined antidepressive therapy modulated signs and symptoms of TMD	Clinical evaluation according to RDC/TMD; EMG; Helkimo's Index criteria
Pelkonen et al. (2013)	Depression	RCS	*N* = 12058		“Parental depression during the offspring's childhood associated significantly with facial pain and with TMJ pain at jaw rest”	SCL-25 DS; Finnish Hospital Discharge Register; postal questionnaire
Yachida et al. (2012)	Depression	PCS	*N* = 115 (66% females)	RDC/TMD	“Significant correlation between EMG activity and depression scores”	Clinical evaluation according to RDC/TMD; ICHD-II; examination for clinical signs or symptoms related to sleep bruxism; McGill Pain Questionnaire; EMG
Mladenović et al. (2014)	Depression, Somatization	CSCCS	*N* = 88 (43% females)	RDC/TMD	Increased depression and somatization levels in MFP patients	Clinical evaluation according to RDC/TMD; SCL-90-R; Helkimo's Occlusal Index; overjet; overbite
Kim et al. (2012)	Depression, mood disorders	PCSS	*N* = 317 (76% female)	RDC/TMD axis II	Higher score on certain items, such as fatigue, difficulty in falling asleep, and helplessness in patients with TMD. Patients with MFP had higher depression scores	Clinical evaluation according to RDC/TMD; clinical and radiological examination
Manfredini et al. (2009)	Depression, Somatization	RCSS	*N* = 96 (78% females)	RDC/TMD	Highest scores in almost all psychometric scales among MFP patients	Clinical evaluation according to RDC/TMD; full version of SCL-R
Shedden Mora et al. (2012)	Depression, Stress-related disorders, Mood disorders	PCS	*N* = 106 (84% females)	RDC/TMD	In MFP patients the EMG burst per episode was related to general somatic complaints, TMD related symptoms, pain intensity, depression, and stress level. Patients with MFP were nearly four times more likely to have a psychiatric disorder than healthy controls	Clinical evaluation according to RDC/TMD; German version of the RDC/TMD Axis II self-report measures; Screening for Somatoform Symptoms; German version of the Center for Epidemiological Studies Depression scale; German version of the Patient Health Questionnaire; EMG
Shibuya et al. (2009)	Depression, Mood disorders	PCSS	*N* = 71 (78% females)	RDC/TMD	Among MFP patients depressive mood correlated significantly with occlusal discomfort, whereas in disc displacement patients occlusal discomfort was promoted by sleep complaints but not depressive mood	Clinical evaluation according to RDC/TMD; HADS, TMD-related Limitation of Activities in Daily Living Questionnaire, occlusal discomfort screening tool
Buenaver et al. (2012)	Depression, mood disorders, sleep disturbances	RCSS	*N* = 214 (74% females)	RDC/TMD	In MFP patients depression scores were significantly correlated with pain severity and interference, catastrophizing, and poorer sleep quality. “The pain catastrophizing was associated with clinical pain severity”	Clinical evaluation according to RDC/TMD; Pain Catastrophizing Scale; Pittsburgh Sleep Quality Index; the Brief Pain Inventory; BDI
Reissmann et al. (2008)	Depression	RCSS	*N* = 491 (78% female)	RDC/TMD	“There are no significant differences between pain location and depression.” Higher depression scores in joint pain patients	Clinical evaluation according to RDC/TMD; MPI; Giessen-test; GCPS

*PCS, prospective cohort study; RCSS, retrospective cross-sectional study; CCS, case-control study; CSCCS, cross-sectional case-control study; RCS, retrospective cohort study; PCSS, prospective cross-sectional study; MFP, myofascial pain; TMD, Temporomandibular Disorders; FM, fibromyalgia; RDC/TMD, Research Diagnostic Criteria For Temporomandibular Disorders; HADS, Hospital Anxiety and Depression Scale; AAOP, American Academy of Orofacial Pain; CID-S, Composite International Diagnostic-Screener; COMT, catechol-O-methyltransferase; IHS, International Headache Society; SCL–90, Symptom Check list 90; GCPS, Graded Chronic Pain Scale; VAS, Visual Analog Scale; PPT, Pressure pain threshold; ACR, American College of Rheumatology; CSQ, Coping Strategies Questionnaire; ICHD-II, International Classification of Headache Disorders; DSM-IV, Diagnostic and Statistical Manual of Mental Disorders; BDI, Beck Depression Inventory; HDRS, Hamilton Depression Rating Scale; HARS, Hamilton Anxiety Rating Scale; MPI, Multidimensional pain inventory; CMI, Craniomandibular Index; SCID, Statistics and Diagnosis of Mental Disorders; SCL -25 DS, Symptom Check list 25 – Depression Scale; STAI, State-Trait Anxiety Inventory; EMG, electromyography; MFIQ, Mandibular Function Impairment Questionnaire*.

### Characteristics of subjects included in the primary studies

The total number of participants included in the studies ranged from 12,058 to 18. In most studies subjects were adults, mainly females, and in five studies the sample was composed only of females. (Slade et al., 2007; Nilsen et al., 2008; Vedolin et al., 2009; Nilsson and Dahlström, 2010; García-Moya et al., 2015). In 70% of studies individuals were assessed based on RDC/TMD as a diagnostic criterion. Other diagnostic criteria were Graded Chronic Pain Scale (Park et al., 2010; Velly et al., 2011), Guideline of diagnosis of TMD by the American Academy of Orofacial Pain (Kindler et al., 2012; Boggero et al., 2016), Diagnostic Criteria for FM (Nilsen et al., 2008; Pfau et al., 2009; Velly et al., 2010; Alfvén, 2012; García-Moya et al., 2015), International Classification of Headache Disorders II (Mongini et al., 2007; de Tommaso et al., 2009), International Headache Society (Glaros et al., 2007), Diagnostic and Statistical Manual of Mental Disorders-IV (Mongini et al., 2007; Vidaković et al., 2016), Craniomandibular Index (Penna et al., 2009), and Craniomandibular Index/RDC (Velly et al., 2011). In three studies only healthy subjects were included and RDC/TMD were treated as excluding criteria (Slade et al., 2007; Komiyama et al., 2008; Manfredini et al., 2011). MMP patients examined in the included studies often suffered from other concomitant disorders, such as FM (Nilsen et al., 2008; Pfau et al., 2009; Velly et al., 2010; Alfvén, 2012; Klasser et al., 2014; García-Moya et al., 2015) and headaches (Glaros et al., 2007; Mongini et al., 2007; de Tommaso et al., 2009).

### Outcome measurement tools

Studies differed in psychometric assessment tools. In all, about 20 different inventories were used to evaluate the mental status of studied individuals. The most common were Hospital Anxiety and Depression Scale, Symptom Check List 90/-R, Beck Depression Inventory, State-Trait Anxiety Inventory, Hamilton Depression and Anxiety Rating Scales, Symptom Check List 25—Depression Scale. Seventy-nine percent of studies concerned depression, 42% anxiety, 29% mood disorders, and 21% stress-related disorders. Other considered mental states were somatization, catastrophizing, sleep disturbances, phobia, paranoia, psychoticism, and hostility.

### Quality assessment

We finally included 38 papers, of which prospective cohort studies were found to be the most common research design. In addition, we considered prospective cohort studies to be the most reliable type of included research. However, there were also many cross-sectional studies. This type of study explores relationships occurring only at one point in time in the appropriate population; thus, no causal relationship can be established.

### Evidence synthesis

The quality of the evidence is presented in Table 2 as overall GRADE score for each primary outcome. Due to the study design (only observational studies, not randomized control trials or systematic reviews) the initial GRADE score of included studies was decreased^1^. Other common causes of score reduction included limited generalizability of the reported results, quality flaws and clinical heterogeneity between studies. Eventually, none of the outcome evidence was judged as high quality.

**Table 2:** **Summary findings for the primary outcomes**.

**Primary outcome**	**Outcome significancy**	**Trials (year)**	**No. of participants (studies)**	**Quality of the evidence (GRADE)**
Anxiety	Significant correlation	Kindler et al. (2012) de Tommaso et al. (2009) Mongini et al. (2007) Manfredini et al. (2011) Komiyama et al. (2008) Klasser et al. (2014) Pizolato et al. (2013) Nifosì et al. (2007) Galli et al. (2009) Nadendla et al. (2014) Guarda-Nardini et al. (2012)	5,802 (eleven studies)	+ + + − moderate due to indirectness
	No significant correlation	Calixtre et al. (2014) Vedolin et al. (2009) García-Moya et al. (2015) Pfau et al. (2009) Giannakopoulos et al. (2010)	382 (five studies)	+ + −− low due to indirectness, imprecision
Depression	Significant correlation	Slade et al. (2007) Park et al. (2010) Velly et al. (2011) Kindler et al. (2012) Boggero et al. (2016) Velly et al. (2010) de Tommaso et al. (2009) Mongini et al. (2007) Vidaković et al. (2016) Penna et al. (2009) Klasser et al. (2014) Galli et al. (2009) Giannakopoulos et al. (2010) Guarda-Nardini et al. (2012) Cioffi et al. (2014) Ivkovic et al. (2008) Pelkonen et al. (2013) Yachida et al. (2012) Mladenović et al. (2014) Kim et al. (2012) Manfredini et al. (2009) Shedden Mora et al. (2012) Shibuya et al. (2009) Buenaver et al. (2012)	21,536 (twenty four studies)	+ + + − moderate due to indirectness
	No significant correlation	Calixtre et al. (2014) Pfau et al. (2009) Manfredini et al. (2011) Pizolato et al. (2013) Nifosì et al. (2007) Reissmann et al. (2008)	725 (six studies)	+ + −− low due to indirectness, imprecision
Mood disorders	Significant correlation	Slade et al. (2007) Nilsson and Dahlström (2010) Velly et al. (2011) Pfau et al. (2009) Nifosì et al. (2007) Kim et al. (2012) Shedden Mora et al. (2012) Shibuya et al. (2009) Buenaver et al. (2012)	1,537 (nine studies)	+ + + − moderate due to indirectness
	No significant correlation	Penna et al. (2009) Manfredini et al. (2011)	75 (two studies)	+ −−− very low due to indirectness, imprecision, inconsistency
Stress-related disorders	Significant correlation	Slade et al. (2007) Vedolin et al. (2009) Nilsson and Dahlström (2010) Alfvén (2012) Glaros et al. (2007) Vidaković et al. (2016) Shedden Mora et al. (2012)	573 (seven studies)	+ + −− low due to indirectness, imprecision
	No significant correlation	Nilsen et al. (2008)	18 (one study)	+ −−− very low due to indirectness, imprecision, inconsistency

*Indirectness – limited generalizability of the reported results*.

*Imprecision – quality flaws*.

*Inconsistency – clinical heterogeneity between studies*.

#### Anxiety

Among the selected studies, 16 mentioned relationships between anxiety and MFP (Mongini et al., 2007; Nifosì et al., 2007; Komiyama et al., 2008; de Tommaso et al., 2009; Galli et al., 2009; Pfau et al., 2009; Vedolin et al., 2009; Giannakopoulos et al., 2010; Manfredini et al., 2011; Guarda-Nardini et al., 2012; Kindler et al., 2012; Pizolato et al., 2013; Calixtre et al., 2014; Klasser et al., 2014; Nadendla et al., 2014; García-Moya et al., 2015). Eleven of these, performed on a population of 5802 subjects, showed this relationship to be significantly correlated (Mongini et al., 2007; Nifosì et al., 2007; Komiyama et al., 2008; de Tommaso et al., 2009; Galli et al., 2009; Manfredini et al., 2011; Guarda-Nardini et al., 2012; Kindler et al., 2012; Klasser et al., 2014; Nadendla et al., 2014). The quality of evidence for this group was moderate. In five studies there was no significant relationship (Pfau et al., 2009; Vedolin et al., 2009; Giannakopoulos et al., 2010; Calixtre et al., 2014; García-Moya et al., 2015) and, due to the limited generalizability of the reported results and methodological flaws, their GRADE score was low.

#### Depression

Among the main outcomes, depression was studied most frequently. Thirty research teams have investigated the relationship between MFP and depression. Twenty-four of these (Mongini et al., 2007; Slade et al., 2007; Ivkovic et al., 2008; de Tommaso et al., 2009; Galli et al., 2009; Manfredini et al., 2009; Penna et al., 2009; Shibuya et al., 2009; Giannakopoulos et al., 2010; Park et al., 2010; Velly et al., 2010, 2011; Buenaver et al., 2012; Guarda-Nardini et al., 2012; Kim et al., 2012; Kindler et al., 2012; Shedden Mora et al., 2012; Yachida et al., 2012; Pelkonen et al., 2013; Cioffi et al., 2014; Klasser et al., 2014; Mladenović et al., 2014; Boggero et al., 2016; Vidaković et al., 2016) have found a significant correlation between these issues and six studies have not (Nifosì et al., 2007; Reissmann et al., 2008; Pfau et al., 2009; Manfredini et al., 2011; Pizolato et al., 2013; Calixtre et al., 2014). The quality of evidence in the first group was moderate due to the limited generalizability of the reported results, while in the second group the score was low.

#### Mood disorders

Among the selected studies, ten investigated the relationship between MFP and mood disorders (Nifosì et al., 2007; Slade et al., 2007; Penna et al., 2009; Pfau et al., 2009; Shibuya et al., 2009; Nilsson and Dahlström, 2010; Park et al., 2010; Manfredini et al., 2011; Buenaver et al., 2012; Kim et al., 2012; Shedden Mora et al., 2012). Most of these found a significant correlation between these issues; however, two of these did not support this assertion (Penna et al., 2009; Manfredini et al., 2011). In the first group, the quality of evidence was moderate due to the limited generalizability of the reported results. In the second group this relationship was scored very low.

#### Stress-related disorders

Among the main outcomes, stress-related disorders seem to be examined least often. Eight studies mentioned this problem (Glaros et al., 2007; Slade et al., 2007; Nilsen et al., 2008; Vedolin et al., 2009; Nilsson and Dahlström, 2010; Alfvén, 2012; Shedden Mora et al., 2012; Vidaković et al., 2016), only one of which did not find any significant correlation between stress and MMF (Nilsen et al., 2008). Moreover, the quality of evidence for this outcome was either low or very low.

## Discussion

### Anxiety

Many research teams have tried to establish whether there is an association between anxiety and pain location (Mongini et al., 2007; Nifosì et al., 2007; Kindler et al., 2012; Klasser et al., 2014). Mongini et al. (2007) found that the prevalence of anxiety was higher in patients with myogenous pain than in those with arthrogenous TMD and neuropathic pain. Researchers concluded that, regardless of the pain localization, anxiety and depression independently increased the likelihood of greater muscle tenderness. Nifosì et al. (2007) reported similar findings. However, comparisons of patients divided in a twofold manner contributed to variations in results. Firstly, the group with a myofascial component scored higher on anxiety, paranoia, psychoticism, hostility subscales, and the global severity index than the group with only articular pain. Then, a similar comparison of the three TMD subgroups separately, i.e., (1) with muscle disorders (2) with painful joint disorder and (3) mixed, found no significant differences. Subjects with muscle involvement presented significantly more frequently with a positive psychiatric history and greater lifetime use of psychotropic drugs than did the other patients. Unfortunately, in both studies there was no healthy control group. Among studies comparing patients suffering from MFP with healthy controls (Galli et al., 2009; Pfau et al., 2009; Pizolato et al., 2013; Nadendla et al., 2014) or the general population (Giannakopoulos et al., 2010), there seems to be no certain convergence between the findings. Some of these studies (Manfredini et al., 2009; Pizolato et al., 2013; Nadendla et al., 2014) showed that patients with MFP scored higher on anxiety than healthy controls. However, there have been investigations (Pfau et al., 2009; Giannakopoulos et al., 2010) that do not support this result. Another two findings that did not show any relation between anxiety and MMF (Vedolin et al., 2009; Calixtre et al., 2014) were conducted among young adults. The first study investigated clinical symptoms and jaw functionality in 19 college students with TMD (Calixtre et al., 2014). The second study assessed the pressure pain threshold of masticatory muscles and the level of pain in 29 female dental students suffering from MMF compared to 16 asymptomatic controls (García-Moya et al., 2015).

There have also been studies relevant to anxiety, MMF and comorbid conditions such as FM (de Tommaso et al., 2009; García-Moya et al., 2015). According to García-Moya et al. (2015), the palpation pain of the masseter was significantly greater in FM patients and the pain intensity and state anxiety was correlated only among FM sufferers. Notwithstanding this fact, no significant differences in anxiety were observed between FM and control group. Unfortunately, the study sample size was limited. de Tommaso et al. showed a comorbidity of FM in tension-type headache (de Tommaso et al., 2009). The total tenderness score was higher in FM patients. FM patients (especially tension-type headache sufferers) scored significantly higher in terms of anxiety and depression.

Two studies investigated relationships between masticatory muscle activity according to anxiety using electromyography (EMG) (Komiyama et al., 2008; Manfredini et al., 2011) and aimed to clarify how psychological aspects affect masticatory muscles activity. Manfredini et al. (2011) reported that an above median value for the temperamental anxiety score, depression and anger was correlated to EMG-assessed non-functional nocturnal masticatory activity. Unfortunately, the study was conducted on an insufficiently representative sample and sleep-related EMG recordings were performed only once. Also, Komiyama et al. (2008) showed that anxiety impacts on the reflexes and pain reactions associated with masticatory muscles.

Two investigations presented that salivary cortisol level was related to anxiety scores among MMF patients, but without any dependences among healthy controls. Galli et al. (2009) showed that after administration of dexamethasone patients with chronic myogenous TMD exhibited enhanced and persisting suppression of cortisol levels. Their psychological evaluation revealed that they scored significantly higher in terms of depression, anxiety, physical and mental fatigue. Nadendla et al. (2014) found that awakening salivary cortisol levels and anxiety scores were significantly greater among patients with MFP (without any other joint symptoms).

### Depression

Many researchers have attempted to establish whether depression episodes influence pain location (Reissmann et al., 2008; Manfredini et al., 2009; Giannakopoulos et al., 2010; Park et al., 2010; Kim et al., 2012; Klasser et al., 2014; Boggero et al., 2016). Most of these have found that patients with muscle disorders have significantly higher depression scores (Manfredini et al., 2009; Park et al., 2010; Kim et al., 2012; Klasser et al., 2014; Boggero et al., 2016). Klasser et al. (2014) showed that patients with myogenous TMD more often reported not only depression, but also anxiety, psychiatric treatment, phobias and severe headaches than arthrogenous TMD. In this study, the psychosocial assessment of the sample was based on a dichotomous medical questionnaire and there was no verification of the accuracy of self-reported co-existing conditions. The absence of healthy controls and a reliable psychometric inventory make comparisons with other studies difficult. Similar findings were reported by Giannakopoulos et al. (2010), who established that patients with MFP were significantly more depressed than patients with joint pain. However, some researchers have not found any significant relationships between depression and muscle pain when comparing three groups of patients suffering from (1) muscle disorder, (2) arthralgia and/or osteoarthritis, and (3) both (Reissmann et al., 2008; Manfredini et al., 2009; Guarda-Nardini et al., 2012). Unfortunately in all these studies, there were no controls to compare with.

Depression was often considered the main factor to be reported in the included studies; however, other factors defined as secondary outcomes were also investigated. Some researchers have studied somatization along with depression (Park et al., 2010; Guarda-Nardini et al., 2012; Kindler et al., 2012; Shedden Mora et al., 2012; Yachida et al., 2012; Cioffi et al., 2014; Mladenović et al., 2014). Mladenović et al. (2014) investigated psychological and dentition-related aspects of TMD in class III patients referred for orthognathic surgery and showed that MFP was related to higher grades of depression and somatization (Mladenović et al., 2014). Similar findings were reported by Shedden Mora et al. (2012), who recorded nocturnal masseter activity and found that in MFP patients one component of nocturnal masseter activity, namely burst per episode, was related to general somatic complaints, TMD related symptoms, pain intensity, depression, and stress level. Patients with MFP were nearly four times more likely to have a psychiatric disorder than healthy controls. Also, Cioffi et al. (2014) found an increased incidence of episodes of depression and somatization in patients with orofacial pain. Catastrophizing was also considered as an issue accompanying MMP. Velly et al. (2010, 2011) found a positive correlation between the intensification of pain symptoms in the 18 months and catastrophizing or depression. One study mentioned post-traumatic stress disorder (Vidaković et al., 2016). This described a higher appearance of MFP syndrome among Croatian soldiers who had taken an active part in war. Fifty-nine percent of the respondents had MFP syndrome, which more often concerned patients with severe and moderate depression.

In some studies, researchers have used EMG to find correlations between muscle activity and depression (Ivkovic et al., 2008; Yachida et al., 2012). Ivkovic et al. conducted a study whose aim was to evaluate the long-term effects of antidepressant therapy on chronic pain and related disability, and masseter silent periods in psychiatric depressive patients with TMD (Ivkovic et al., 2008). The study showed lower occurrence of muscular TMD subtype and chronic muscle pain in patients on antidepressant therapy protocols. Also, Yachida et al. compared the EMG activity of masseter muscles during sleep in patients with orofacial pain and in a control group and found a positive correlation between increased myogenic activity of facial muscles and depression (Yachida et al., 2012).

### Mood disorders

Along with mood disorders, research teams have studied sleep disturbances and their influence on participants' habitual life (Shibuya et al., 2009; Buenaver et al., 2012). Shibuya et al. (2009) reported that in patients with MFP a depressive mood was common and this aggravated occlusal discomfort and sleep complaints. Buenaver et al. (2012) reported similar findings. Pain catastrophizing, defined as a “negative cognitive-affective response to pain involving rumination, helplessness, and magnification,” was strongly correlated with increased levels of pain severity and sleep disturbances. Other scientists have also studied muscle pain as a possible result of mood disorders. Pfau et al. investigated psychological parameters among TMD myogenic patients, FM patients and healthy controls (Pfau et al., 2009). Higher levels of pain, catastrophizing, disability index (but not anxiety and depression) were found in TMD patients compared to healthy individuals. Nilsson and Dahlström (2010) found that there were no differences in fatigue, emotional distress, cognitive symptoms, or somatic symptoms between patients with different TMD diagnoses. Nonetheless, the psychometric scale scores were significantly higher among TMD patients than in controls.

### Stress-related disorders

Some researchers investigated the impact of emotional distress on pain sensitivity and muscle tenderness (Slade et al., 2007; Vedolin et al., 2009; Alfvén, 2012). According to Slade et al. (2007), stress, and also depression and mood disorders were associated with pain sensitivity and were qualified as risk factors of TMD. Vedolin et al. (2009) reported that during stressful periods, the pressure pain thresholds of masticatory muscles in MFP subjects were significantly lower than in controls. Notwithstanding the fact, that both groups were equally anxious and depressed, the MFP individuals had lower pain tolerance. Scientists have reasoned that the external stressor, that is academic examination, had a potential influence on masticatory muscle tenderness. A study conducted among children experiencing psychosomatic pain showed that there was a specific pattern of stress tender points, different from that found in FM sufferers (Alfvén, 2012). A stress tender point localized near the muscle-tendon junction in the temporal region was found only in patients suffering from prolonged stress.

Glaros et al. raised the problem of headache and TMD overlap (Glaros et al., 2007). Participants suffering from headache were significantly more likely to be diagnosed as having MFP, more tension in the jaw, face, head, and stress than those in the non-headache control group.

There was one study employing a salivary cortisol level assay (Nilsson and Dahlström, 2010). It was reported that there were no differences in awakening salivary cortisol levels between TMD patients and the healthy control group and the same held for cortisol levels and emotional distress. Nonetheless, the psychometric scale scores were significantly higher among TMD patients than in controls. An important drawback of the study is the single collection of salvia samples.

### Limitations of the study

The studies included in our systematic review are very heterogeneous. Inconsistences arising from many sources greatly impeded systematic comparisons; thus, we conducted a “narrative, qualitative summary,” as is recommended (Shamseer et al., 2015). Discrepancies related to differences in primary diagnostic criteria employed in the studies were relatively slight, but even if the diagnostic criteria were similar, the structure of comparison groups differed considerably. Some researchers divided their subjects into two (e.g., with myogenous and arthrogenous TMD; Klasser et al., 2014), three (e.g., with MFP, with joint pain and mixed; Park et al., 2010) or even four groups, when the control group was included (Giannakopoulos et al., 2010). Next, they differed in psychometric assessment tools. In all, about 20 different inventories were used to evaluate the mental status of studied individuals. Finally, the studies often differed in their design, sample strength, age and health status of enrolled participants. Thus, the evidence cannot be considered as definitively confirming a cause-and-effect relationship. However the coexistence of anxiety, depression, mood and stress-related disorders among the MMP patients, which is an indisputable scientific fact, must be taken into consideration. Clinicians' improved knowledge and awareness of this multifaceted problem would facilitate an appropriate approach to patients and the making of accurate therapeutic decisions.

## Conclusions

The study of the concomitance of masticatory muscle pain with psychological variables requires careful statistical analysis before any clear conclusions about causality can be made. Unfortunately, in the light of the analyzed literature the causal relationship between mental status and MMP is still not clearly established. As a consequence, no clear explanation of any correlation between masticatory muscle pain and common psychological states, such as anxiety, depression, stress, and disturbances in mood, can be provided. Although the studies consider this question, the researchers usually prove only the co-occurrence of these mental states and dysfunctions of the masticatory muscles among the group of patients, typically in women. The evidence synthesis for main outcomes revealed that studies showing a positive relationship between particular psychological states and masticatory muscle pain provided higher GRADE score than investigations not showing any such relationship. We still need further, properly planned long-term clinical trials and laboratory tests to conclusively prove and describe the pathomechanism inducing masticatory muscle pain in terms of psychological variables.

## Author contributions

MW created a review concept, wrote and edited the manuscript. NG and JS made articles selection, wrote and edited the manuscript. MZ and DZ edited the manuscript and finally revised it before submission. All authors read and approved the final manuscript.

### Conflict of interest statement

The authors declare that the research was conducted in the absence of any commercial or financial relationships that could be construed as a potential conflict of interest.

## Footnotes

^1^http://clinicalevidence.bmj.com/x/set/static/ebm/learn/665072.html

## Abbreviations

MMP: masticatory muscle pain
RDC/TMD: Research Diagnostic Criteria for Temporomandibular Disorders
MFP: Myofacial pain
TTH: Tension-type headache
FM: Fibromyalgia
MeSH: Medical Subject Headings
TMD: Temporomandibular Disorders
TMJ: Temporomandibular Joint
CMI: Craniomandibular Index
TMJD: Temporomandibular Joint Disorders
EMG: Electromyography.
